# Musical experience relates to functional connectivity in older adults

**DOI:** 10.1093/geroni/igab046.2654

**Published:** 2021-12-17

**Authors:** Meishan Ai, Timothy Morris, Laura Chaddock-Heyman, Psyche Loui, Susan Whitfield-Gabrieli, Charles Hillman, Edward McAuley, Arthur Kramer

**Affiliations:** 1 Northeastern University, Brookline, Massachusetts, United States; 2 Northeastern University, Boston, Massachusetts, United States; 3 University of Illinois at Urbana-Champaign, Urbana, Illinois, United States; 4 University of Illinois at Urbana-Champaign, Boston, Massachusetts, United States

## Abstract

Previous studies have shown that engaging in musical activities throughout the lifespan may buffer age-related decline in auditory and motor function, as well as in general cognitive function. MRI studies have demonstrated that individuals with musical training and experience exhibited greater grey matter volume and functional connectivity in extensive brain regions, especially in auditory and motor systems, compared to matched controls with no particular musical training or experience. Therefore, musical activity is a potential protective factor for brain health across lifespan. However, how lifespan musical experience shapes functional connectivity in older adults is still unknown. The current analysis investigated whether general musical experience (Goldsmith Music Sophistication Index) is associated with functional connectivity in older adults (age=65.7±4.4, n=69), focusing on seed regions in primary motor areas (bilateral precentral gyrus) and primary auditory regions (bilateral anterior/posterior superior temporal gyrus) and their functional connectivity towards other areas throughout the whole brain. We found that older adults with more musical experience showed greater functional connectivity between anterior superior temporal gyrus and insula (R2=0.10, p=0.01), and between posterior superior temporal gyrus and cerebellum (R2=0.08, p=0.02). However, musical experience and music-related functional connectivity was not significantly correlated with general cognitive functions in our sample. Overall, our findings suggest that older adults with more musical experience might be more efficient in some aspects of auditory processing and auditory-motor skills, but this may not transfer towards domain-general cognitive tests. Our results support the notion that even non-professional engagement in musical experiences may afford benefits to the aging brain.

